# The Regulatory Role of KIBRA and PTPN14 in Hippo Signaling and Beyond

**DOI:** 10.3390/genes7060023

**Published:** 2016-05-27

**Authors:** Kayla E. Wilson, Nuo Yang, Ashley L. Mussell, Jianmin Zhang

**Affiliations:** Department of Cancer Genetics, Roswell Park Cancer Institute, Buffalo, NY 14263, USA; kayla.wilson@roswellpark.org (K.E.W.); Nuo.yang@roswellpark.org (N.Y.); ashley.mussell@roswellpark.org (A.L.M.)

**Keywords:** KIBRA, PTPN14, Hippo signaling pathway

## Abstract

The Hippo signaling pathway regulates cellular proliferation and survival, thus exerting profound effects on normal cell fate and tumorigenesis. Pivotal effectors of this pathway are YAP/TAZ, transcriptional co-activators whose dysfunction contributes to the development of cancer. Complex networks of intracellular and extracellular signaling pathways that modulate YAP and TAZ activities have recently been identified. Among them, KIBRA and PTPN14 are two evolutionarily-conserved and important YAP/TAZ upstream regulators. They can negatively regulate YAP/TAZ functions separately or in concert. In this review, we summarize the current and emerging regulatory roles of KIBRA and PTPN14 in the Hippo pathway and their functions in cancer.

## 1. Introduction

Hippo signaling plays an important role in the acquisition of certain diseases, including cancer. The purpose of this kinase cascade is to lead to the phosphorylation and eventual cytoplasmic sequestration of two effector molecules, yes-associated protein (YAP), and its paralog WW domain-containing transcription regulator 1 (WWTR1/TAZ). In mammals, the main proteins that are known to directly phosphorylate YAP/TAZ are the large tumor suppressor kinases 1 and 2 (LATS1/2) [1,2,3,4]. If the pathway is dysfunctional and YAP/TAZ are allowed to move into the nucleus, they will bind to a number of transcription factors, most notably the TEA domain (TEAD) family members. These transcription factors will lead to the activation of various genes, which contributes to the proliferative and anti-apoptotic effects that are common drivers of cancer progression [5,6,7,8,9,10,11,12].

Hippo signaling has been heavily studied over the past decade. More and more, focus is switching from the core kinase cascade to uncovering upstream regulators. Two such proteins are Kidney and Brain Protein (KIBRA) and protein tyrosine phosphatase, non-receptor type 14 (PTPN14). Looking at the earlier findings related to both factors, we should not be surprised that they could also be playing roles in the Hippo signaling pathway and its function as a tumor suppressor pathway. Therefore, it is worthwhile to understand the accumulated data available describing the functioning of these proteins in various settings, in order to fully appreciate their relationship to the Hippo pathway.

## 2. KIBRA and PTPN14

In 2003, KIBRA (WWC1) was isolated as a novel cytoplasmic protein with two amino-terminal WW domains, internal C2-like domain, and a carboxy-terminal acid-rich stretch, in a yeast two-hybrid screen [13]. It was named for its enriched expression primarily in the kidney and brain, and is a member of the evolutionarily-conserved WWC family of proteins [14]. Not surprisingly, this localization has led to an abundance of research on KIBRA’s role in mental illnesses, such as schizophrenia and depression; yet it has also been found to be a major player in multiple cell signaling pathways, eliciting a variety of effects on the functioning of both normal and cancerous cells.

PTPN14 was first identified and named Pez in 1995 when a group isolated cDNAs from normal breast tissue and breast tumor cells. Expression of Pez was later reported in a number of other human tissues, including kidney, skeletal muscle, lungs, and placenta [15]. In its early days, PTPN14 was also studied as PTP36 or PTPD2. PTP36, the murine homologue of Pez, became the focus of the earliest studies involving cell adhesion shortly after its identification.

## 3. KIBRA and PTPN14 in Hippo Signaling

The first connection of Kibra to Hippo signaling came through a genetic screen for oocyte polarity mutants in *Drosophila* [16]. Among these was KIBRA, whose loss was found to induce defects in Notch signaling, similar to those previously seen for the canonical Hippo pathway mutants [17,18,19]. Loss of KIBRA was further found to promote a tissue overgrowth phenotype, indicative of defects in Hippo signaling. Importantly, KIBRA forms a complex with Merlin and Expanded, two known activators of Warts (Wts) phosphorylation, to synergistically induce further phosphorylation. This protein complex can also directly bind to Hippo (Hpo) and Salvador (Sav), thus phosphorylating Yorkie (Yki).

KIBRA’s role in Hippo signaling was further characterized in mammalian cells shortly after characterization in *Drosophila* [20]. Studies revealed its regulation of the core cascade through direct interaction with LATS1/2 via its WW domains and stimulation of their phosphorylation. As a result of this increase in LATS phosphorylation, phosphorylation of YAP was enhanced upon overexpression of KIBRA. Such effects were shown to be independent of the mammalian sterile 20-like kinases 1 and 2 (Mst1/2), the upstream kinases normally responsible for the LATS phosphorylation. KIBRA can also upregulate and stabilize the LATS2 protein. Finally, KIBRA was also demonstrated to be a transcriptional target of the Hippo pathway, in that KIBRA mRNA levels were upregulated in both mouse liver and cell lines overexpressing YAP.

KIBRA phosphorylation was characterized relatively recently and was found to be cell cycle-dependent, with high phosphorylation during mitosis [20]. This phosphorylation is mediated by Aurora kinases at Ser539, which are known to play an important role in mitotic control. KIBRA can then be dephosphorylated by protein phosphatase 1 (PP1), previously shown to do so with other Aurora phosphorylated proteins in mitosis. The above processes are connected to Hippo signaling, as phosphorylation of KIBRA at Ser539 is required for its association with neurofibromin 2 (Nf2/Merlin).

The idea that some Aurora kinase substrates can also activate the original kinase was exploited to gain a deeper understanding of how KIBRA works together with them in mitosis [21]. Indeed, KIBRA is not only phosphorylated by Aurora, but it also has the ability to indirectly lead to Aurora phosphorylation. This phosphorylation is in fact required for the full activation of Aurora kinases during mitosis. Aurora A is known to phosphorylate Ser83 of LATS2 and KIBRA overexpression can promote this activity. The relevance of a KIBRA-Aurora-LATS2 axis for control of mitosis is supported by the fact that knockdown of KIBRA has the ability to cause mitotic abnormalities.

The role of KIBRA in cell cycle progression was further expanded to show that it can also be phosphorylated by cyclin-dependent kinase 1 (CDK1) at Ser542 and Ser931 during spindle-stress-induced mitosis [22]. Cell division cycle 14 (CDC14) phosphatases, which control mitotic exit, were also found to associate with and dephosphorylate KIBRA. Together, these proteins are able to regulate cell cycle progression in response to stress. Notably, this function of KIBRA is independent of Hippo signaling, with phosphorylation levels at these sites having no effect on YAP phosphorylation.

PTPN14 came onto the Hippo signaling scene in 2012 when several groups published work connecting it to the Hippo effector YAP1 [23,24,25]. PTPN14 was identified as a possible tumor suppressor through shRNA-mediated loss-of-function screen. It was found to exert this function through an interaction with YAP1, ultimately suppressing cell proliferation through promotion of cell density dependent YAP1 cytoplasmic translocation and independent from PTPN14 phosphatase activity [25]. In parallel, we have identified the YAP1 and PTPN14 interaction through immunoprecipitation (IP) mass spectrometry analysis. This interaction is facilitated by PTPN14’s PPxY motifs and YAP1’s WW domains [23]. Furthermore, downregulation of PTPN14 phenocopies YAP1 overexpression in mammary epithelial cells, inducing oncogenic transformation [23,24,25]. This relationship between YAP and PTPN14 was also found to contribute to the phenotype of chemotherapeutic agent resistance present in the ovarian cancer cells overexpressing YAP [26]. Knockdown of YAP expression increased the cell response to the EGFR inhibitor erlotinib. Meanwhile, the PPxY motifs of PTPN14 mediated binding to YAP and subsequently reduced its transcriptional activity. It was then consistently observed that expression of a PPxY-containing fragment of PTPN14 rendered cells more sensitive to erlotinib.

When a group carried out the affinity purification-mass spectrometry (AP-MS) to identify new binding partners of Kibra in *Drosophila*, they identified the link between KIBRA and PTPN14 (Pez) [27]. Researchers identified Pez and characterized its function and relationship to Hippo signaling in adult midgut homeostasis. Although specific implications of this KIBRA-Pez interactions were not yet known, Pez was established as an upstream component of the Hippo pathway, where it works to restrict the transcriptional activity of Yki (YAP) and thus growth in the flies.

In mammalian cells, we have demonstrated that KIBRA interacts with PTPN14, and along with LATS1, forms a trimeric complex [28]. PTPN14 and KIBRA can activate LATS1 independently or cooperatively, independent of the Mst1/2 proteins. Knockdown of PTPN14 decreased the pLATS1 and pYAP levels, which led to nuclear YAP localization and subsequent transcription of YAP target genes involved in malignant phenotypes such as increased cell migration and aberrant acini formation. Notably, overexpression of KIBRA significantly reduced these phenotypes, underlining the important interactions among these factors in this setting.

## 4. Other Interaction Partners and Functions of KIBRA and PTPN14

Over the years, many different KIBRA-interacting proteins have been identified, each with its own implications in cell biology. The first of these was Dendrin, which was used in the original yeast two-hybrid screen for KIBRA’s characterization [13]. The same group also identified protein kinase Cζ (PKCζ), a member of the atypical protein kinase C (aPKC) subfamily usually known for its roles in synaptic plasticity and memory formation [29,30]. By interacting with and phosphorylating KIBRA’s S975/S978 residues, it was speculated that PKCζ could be helping to regulate KIBRA dimer formation, ultimately regulating its other cellular functions. KIBRA’s relationship with atypical protein kinase Cs (aPKCs) and cell polarity was further fleshed out to show that KIBRA has the ability to regulate epithelial cell polarity through a suppression of apical exocytosis. It does so by directly inhibiting the aPKC kinase activity [31]. Additionally, this KIBRA-aPKC interaction has recently been implicated in the regulation of the starvation-induced autophagy in *Drosophila* [32].

Another KIBRA-interacting protein was found through a search for novel dynein light chain 1 (DLC1), a cytoskeletal signaling component, interactors [33]. DLC1 was known to interact with and trans-activate the estrogen receptor (ER) and assist in stimulating the growth of breast cancer cells [34]. The complex formed by the interaction of DLC1 and KIBRA was found to be recruited to ER-responsive promoters and this is required for DLC1 to transactivate ER. Interestingly, KIBRA can also interact with histone H3, putting forth a model of a complex responsible for ER transactivation.

Endosomal sorting is another cellular process in which KIBRA plays a role [35]. Sorting nexin 4 (SNX4), which is involved in intercellular trafficking, is able to interact with KIBRA and coordinate carrier transport between the early endosome and the endocytic recycling compartment. 

When considering that KIBRA was previously found to be involved with the transactivation of ER and the stimulation of breast cancer cell growth, it is reasonable to look into the role it may play in mammary gland development. In a prolactin *Prl*^−/−^ model, KIBRA was found to be decreased in the mammary glands that had impaired development and its expression level was also related to specific developmental events [36]. In pregnancy, KIBRA is upregulated, followed by a decrease during lactation. Expression then rises again during involution after weaning. Clearly, KIBRA plays a role in these processes; so to understand its mechanism of action, a bioinformatics approach was used to identify potential binding partners. Discoidin domain receptor 1 (DDR1), a collagen activated tyrosine kinase receptor, was one such partner. KIBRA can bind to DDR1 and is then released in the presence of its ligands, collagen type I or IV. Through this interaction and release, KIBRA can regulate collagen-stimulated extracellular signal-related kinase/mitogen-activated protein kinase (ERK/MAPK) activation and cellular proliferation in the developing mammary gland.

In another yeast two-hybrid screen using a podocyte cDNA library with the polarity protein Pals1-associated tight junction protein (PATJ) as bait, KIBRA was identified to be a PATJ interactor [37]. KIBRA knockdown was able to impair directed cell migration in this system. Along with PATJ, KIBRA also interacts with dendrin and synaptopodin, providing a link between cytoskeleton and polarity proteins to regulate cell motility of podocytes.

Since KIBRA was previously shown to be a substrate of CDC14, another group examined the relationship between these two proteins and the actin cytoskeleton [38]. KIBRA and human CDC14A (hCDC14A) were found to co-localize at the cell leading edge in order to regulate cell mobility and adhesion. While cells without hCDC14A activity had increased migration and altered adhesion behavior, the overexpression of KIBRA was able to rescue these phenotypes. These studies indicated that KIBRA is working downstream of hCDC14 in the same pathway, because there was no additive effect seen when both proteins were overexpressed.

Very recently, KIBRA has been found to interact with ataxia telangiectasia mutated (ATM), whose phosphorylation at T1006 is necessary for optimal DNA double-strand break repair in cancer cells [39]. Without KIBRA, cells are not able to overcome and repair DNA damage effectively.

Finally, studies have been performed to understand the gene expression regulation of KIBRA [40]. Its expression was found to be regulated by three independent promoters, which are cell-type-specific. In kidney cells, transcription factor 7-like 2 (TCF7L2) is able to interact with the KIBRA promoter, in a transcriptional module with β-catenin, YAP1, and TEAD, to drive KIBRA transcription. The many functions of KIBRA that have been described show several different roles, depending on the context. KIBRA can serve as either a positive or negative regulator of cell behaviors, such as migration and proliferation.

Though not as extensive, there is also work describing PTPN14’s other interaction partners and functions. Using an inducible PTP36 Hela cell line, overexpression of PTP36 was seen to play a role in a reduction of the ability of cells to spread and grow, as well as in decreasing the number of observable actin stress fibers and focal adhesions [41]. In another study from the same group, PTP36’s relationship with cell-substrate adhesion was explored [42], as it was rapidly dephosphorylated when cell-substrate adhesion was disrupted and then rephosphorylated again after actin polymerization and reattachment of the cells.

In 2000, Pez was shown to also have a nuclear function, leading to the induction of cell proliferation [43]. Localization of Pez to the nucleus in endothelial cells was regulated by cell density and serum concentrations. Cells that were either in confluent monolayers or that were serum-starved had cytoplasmic Pez, whereas proliferative cells or those re-fed with serum after starvation had nuclear Pez. This nuclear localization was also found to be regulated by TGFβ, with its overexpression inhibiting cell proliferation and keeping Pez in the cytoplasm. This study demonstrated a new role for PTPN14 in the nucleus and suggested that, at least in endothelial cells, they are highly proliferative. 

The same group later focused on the localization of Pez to intercellular junctions that they found to occur in cells in quiescent monolayers [44]. Pez was able to co-localize with E-cadherin at adherens junctions, and was a regulator of β-catenin via directly affecting its phosphorylation status. Dominant-negative Pez was also able to enhance cell motility, which is related to the fact that when adherens junction proteins have increased tyrosine phosphorylation.

Although PTPN14 was previously shown to decrease cell behaviors such as migration, it was interestingly shown to be able to induce EMT in certain settings [45]. In organogenesis, PTPN14 played a role in inducing the TGFβ signaling for developmental EMT. TGFβ3, in particular, was co-expressed with Pez in a number of developing tissues in zebrafish.

## 5. Expression and Involvement in Cancer Cell Signaling

Due to KIBRA and PTPN14’s functions in many cell signaling activities that are involved in the development and progression of cancer, along with their seemingly tumor suppressive roles in the Hippo pathway, interest has been building to understand how expression of these proteins might be directly involved in cancer.

In osteosarcomas, repression of KIBRA is linked to the ability of cancer cells to maintain their stemness [46]. Researchers were able to demonstrate that the transcription factor sex determining region Y-box 2 (Sox2) could maintain cancer stem cells in these cancers. Specifically, through gene expression analysis of Sox2-regulated genes, it was found that several Hippo pathway-related genes, including Nf2 (Merlin), WWC1 (KIBRA), and YAP, are deregulated in osteosarcomas. The cancer stem cell population in this disease type are marked by the repression of Merlin and KIBRA and high YAP expression, with the opposite being true for more differentiated tumors. Sox2’s ability to bind to, and negatively regulate, KIBRA, along with NF2, demonstrates the importance of upstream regulation of Hippo signaling.

Methylation status of the KIBRA gene has also been reported to be a prognostic indicator in chronic lymphocytic leukemia [47]. In a cohort of 95 CLL patients, 35% had frequent KIBRA methylation and were associated with unfavorable biological prognostic parameters, including high CD38 expression. This data, along with data supporting the deregulation of other Hippo pathway members [48], indicate a role for Hippo signaling in B cell malignancies.

Functional studies of KIBRA’s activity in breast cells shed light on the importance of its regulatory roles [49]. When KIBRA is knocked down in MCF10A cells, it induces an epithelial-mesenchymal transition (EMT) phenotype, similar to what is seen from the overexpression of YAP, as demonstrated by cell morphology, a switch from epithelial to mesenchymal markers, growth in soft agar, and increased cell migration. As seen previously in other studies, these features are dependent on LATS, but not Mst. Furthermore, the expression of KIBRA is able to antagonize YAP-induced EMT and transcriptional regulation. Most importantly, low expression of KIBRA, along with an increased YAP/TAZ target connective tissue growth factor (CTGF) expression, was found to correlate with claudin-low breast cancer cell lines, as well as in claudin-low primary breast tumors, which, generally, are aggressive and have a poor prognosis.

Due to the finding that overexpression of aPKC is associated with poor prognosis in gastric cancer and its previous link to KIBRA, a group studied their relationship in gastric cancer [50]. In the 164 patient tissues examined, there was a relationship between the expression of aPKC and KIBRA, where high KIBRA expression in low aPKC expressing gastric cancers led to a poor prognosis and shorter disease-free survival. Importantly, KIBRA expression by itself did not correlate to survival, but only in conjunction with low aPKC, showing that KIBRA is causing a loss of aPKC activity, loss of polarity, and increased invasiveness. 

After the previous findings, groups became interested in the possible relationship of PTPN14 to other signaling pathways involved in the maintenance of cancer cells. Using a phospho-proteomic approach, potential novel substrates of PTPN14 were found, including breast cancer anti-estrogen resistance protein 1 (p130Cas) [51]. p130Cas by itself has been thought to play a role in tumorigenesis, with overexpression in a subset of breast cancers [52], leading to tamoxifen resistance [53]. p130Cas is a direct substrate of PTPN14, which can specifically dephosphorylate it at tyrosine residue 128 (Y128), the same site that is prone to phosphorylation by SRC proto-oncogene, non-receptor tyrosine kinase (Src). Colorectal cancer cells that have higher levels of pY128 p130Cas are more susceptible to treatment with the Src family kinase inhibitor, Dasatinib. Additionally in this model, epidermal growth factor (EGF), whose signaling plays a critical role in colorectal cancer tumorigenesis, can stimulate p130Cas Y128 phosphorylation. Overall, levels of pY128 p130Cas, which could be favorably decreased by PTPN14, could be a predictive marker for patients’ response to Dasatinib treatment in colorectal cancer. 

MicroRNAs (miRNAs) play an important role in the development of intrahepatic cholangiocarcinoma (ICC). miR-21, of which PTPN14 is a direct and functional target, was found to be significantly upregulated in ICC patient serum [54]. PTPN14 was found through multiple miRNA prediction algorithms and verified using luciferase reporter assays to show that miR-21 significantly repressed activity of reporter vectors with wild type PTPN14. Additionally, mRNA and protein levels of PTPN14 were increased when miR-21 expression was inhibited, whereas the level of YAP expression was decreased in this setting. These findings were supported by gain- and loss-of-function studies showing that PTPN14 overexpression could mimic miR-21 inhibition and PTPN14 silencing could rescue the effects of miR-21 inhibitors on ICC cells. Finally, in ICC patient samples, high miR-21 expression was related to poor prognosis, whereas miR-21 and PTPN14 were inversely correlated.

In breast cancer, PTPN14 has the ability to inhibit metastasis through the alteration of protein trafficking [55]. For example, in a xenograft breast cancer model, knockdown of PTPN14 in triple-negative breast cancer cells was able to promote invasiveness and metastasis. This could be traced to the fact that PTPN14 has the ability to suppress the secretion of prometastatic factors when the medium from shPTPN14 cells was injected into the peritoneum of mice, resulting in increased growth promotion and metastasis. Upon loss of catalytically-functional PTPN14, there was an increase in the secretion of growth factors, such as interleukin 8 (IL-8). This study also identified protein kinase C, delta (PRKCD), and Ras and Rab interactor 1 (RIN1), which are involved in receptor trafficking, as PTPN14 substrates. While PTPN14 is mutated in a number of cancers, increased PRKCD and RIN1 expression correlated with decreased overall survival in breast cancer, with the PRKCD correlation significant in the luminal A subtype.

In mammary epithelial cells, PTPD2 (PTPN14) is connected to erb-b2 receptor tyrosine kinase 2 (ERBB2) signaling [56], of which ERBB2 has been shown to be overexpressed or amplified in a portion of breast cancers and plays a role in tumorigenesis. PTPD2 was identified in a loss-of-function screen of protein tyrosine phosphatases (PTPs) in combination with growth in three-dimensional culture as being able to significantly decrease the multiacinar phenotype that AP150-induced ERBB2 signaling can create. In these 3D cultures, knockdown of PTPD2 enhanced apoptosis and inhibited ERBB2-mediated loss of polarity and lumen filling, while attenuating ERBB2 effector pathways. Conversely, overexpression of PTPD2 increased and enhanced the multiacinar phenotype of the cells. Interestingly in this case, knockdown of YAP was not able to recapitulate this phenotype, indicating that PTPD2 is acting through ERBB2 signaling. PTPD2’s action here can also be activated by the lipid second messenger phosphatidic acid (PA), specifically binding to PTPD2 and increasing its catalytic ability.

Over the past few years, studies have accumulated relating PTPN14 to various cancer types including colorectal cancer, pancreatic cancer, neuroblastoma, and basal cell carcinoma [57,58,59,60]. In addition to the cancer cell signaling work that has been done, genetic profiling is also supporting PTPN14’s emerging role as a tumor suppressor. The first of these linking PTPN14 to cancer began with a screen of the coding exons of all 87 members of the PTP super family in 18 colorectal cancers [60]. PTPN14 was one of the six genes with somatic mutations identified and further analyzed in an additional 157 colorectal cancers, of which 6% had mutations, setting the stage for the study of PTPs in other cancer types.

Transcriptional profiling of fresh frozen tissue derived from primary tumor, tumor invasion front and liver metastases, after orthotopic implantation of the human pancreatic cancer cell line MiaPaca-2 in severe combined immunodeficiency (SCID) mice, revealed differential PTPN14 expression [58]. In the primary tumor, there was high expression of PTPN14, but this was significantly lower in the liver metastases, suggesting a role of PTPN14 in the metastatic process of pancreatic cancer.

In neuroblastoma, whole genome sequencing and gene set enrichment analysis (GSEA) in paired samples at diagnosis and relapse identified inactivating mutations in PTPN14 at relapse, along with a downregulation in genes known to be transcriptionally silenced by YAP [59]. Most recently, genetic profiling of 293 basal cell carcinomas (BCCs) found PTPN14 to be mutated in 23% of the cases [57]. This was related to the previous observation that YAP1 is localized to the nucleus in a number of BCCs. Further confirming this relationship, immunostaining in PTPN14-mutated tumors showed YAP1 nuclear accumulation, whereas tumors with wild-type PTPN14 exhibited a more diffuse YAP1 staining specific to the cytoplasm.

## 6. Conclusions

We have seen the importance of the Hippo signaling pathway in development and organ size control, and particularly in cancer development and progression. As the roles of Hippo signaling have been expanding, it has become critical to uncover further regulatory mechanisms that could link the pathway to others and provide new avenues to explore for promising therapeutic strategies. This is made possible once we appreciate the potential for regulatory mechanisms exerting their effects through multiple overlapping upstream pathways.
